# Social medical insurance system and self-rated health: medical service utilization as the mechanism of action

**DOI:** 10.3389/fpubh.2025.1581130

**Published:** 2025-06-19

**Authors:** Jian Zhou, Mingjing Li, Yucui Lv

**Affiliations:** School of Public Administration, Jinan University, Guangzhou, China

**Keywords:** urban and rural residents basic medical insurance, urban employee basic medical insurance, self-rated health, healthcare utilization, health equity

## Abstract

**Background:**

The fragmented segmentation of the health insurance system has led to differences in healthcare utilization and health outcomes among enrollees with different types of health insurance. This study aims to evaluate the impact of different health insurance system and further explore the pathways of health insurance.

**Methods:**

Using data from the Chinese Family Panel Studies (CFPS) conducted by the China Social Science Survey Center of Peking University in 2018 and 2020, this study employed logit regression model to estimate the impact of different types of health insurance systems on health outcomes. Additionally, healthcare utilization was introduced as a mechanism variable for analysis.

**Results:**

The findings indicate that Urban and Rural Residents Basic Medical Insurance (URRBMI) does not significantly improve health outcomes. In contrast, Urban Employee Basic Medical Insurance (UEBMI) significantly enhances the health status of insured individuals. The influence of medical insurance systems on health exhibits heterogeneity, with education level and regional disparities significantly affecting the effectiveness of these systems. Patterns of healthcare utilization, including inpatient and outpatient medical expenditures and the use of large hospitals, play a crucial role in enhancing the health of insured individuals under UEBMI.

**Conclusion:**

There are significant differences in the impact of various medical insurance systems on the health of insured individuals. UEBMI demonstrates a superior effect in improving the health of insured individuals compared with URRBMI. Future efforts should focus on enhancing the overall planning and coordination of medical insurance, narrowing benefit disparities, and promoting the implementation of a tiered medical system.

## 1 Introduction

Health, as a fundamental human right, is the cornerstone of socioeconomic development and the wealth of countries. Since the founding of New China, the country has developed a multi-tiered health insurance system that includes Urban Employee Basic Medical Insurance (UEBMI) and Urban and Rural Residents Basic Medical Insurance [URRBMI; integrated with the Urban Resident Basic Medical Insurance (URBMI) and New Rural Cooperative Medical System (NRCMS)]. Over time, China has developed a comprehensive system centered on UEBMI and URRBMI, supplemented by critical illness insurance, supplementary medical insurance, and social assistance. This multi-level medical security framework alleviates the financial burden on patients through the effective operation of medical insurance funds, transforms health protection from an individual to a societal responsibility, and aims to improve accessibility and formality in healthcare utilization. Furthermore, this system seeks to improve public health outcomes by increasing access to and formalizing healthcare services (1).

Studies have shown that health insurance plays an important role in improving population health by spreading the risk of disease. Although research findings vary, most studies support the positive impact of health insurance on health. Medicare reduces health disparities among low-income groups (2) and increases the utilization of specialty care services to enhance health (3, 4). The research by Mugo et al. indicates that health insurance reduces mortality, thus having a beneficial impact on promoting health (5). This finding is corroborated by numerous studies in China, which show that overall, social health insurance significantly promotes better health among the older adult (6), UEBMI significantly improves both short- and long-term health of enrollees (7), and URRBMI enhances the population's subjective sense of fairness by enhancing physical and mental health (8), also reducing the gap in health outcomes between rural and urban populations (9). However, Hu found that UEBMI does not have a significant effect on health improvement (10). Zhang et al. indicated that NRCMS has limited impact on enhancing the health of the rural labor force (11).

Medical insurance primarily influences health through healthcare utilization. However, there is no consensus on whether increased healthcare utilization necessarily leads to better health outcomes, as proxy variables for healthcare utilization vary across studies. Research indicates that health insurance improves healthcare utilization. Manning et al. found that in the US, health insurance significantly increases the use of outpatient and inpatient services (12); the districts with higher access to public healthcare services enjoy better health-related outcomes (13). Chinese studies have also confirmed that higher levels of health insurance coverage correlate with greater medical service utilization and improved health status (14). Social health insurance alleviates the problem of expensive and difficult access to medical care, thereby improving public health by increasing both the utilization and accessibility of medical services (15–17).

As the health insurance system achieves broad coverage, the issue of “fragmentation” within the system is becoming increasingly prominent. This fragmentation results in varying impacts of different types of health insurance on health outcomes. Studies have shown that UEBMI and URBMI did not significantly influence healthcare behavior among the insured, while NRCMS significantly increased healthcare utilization. UEBMI led to higher healthcare expenditure, URBMI had a relatively minor impact on healthcare costs, and NRCMS healthcare expenditures for the insured. The positive impact of UEBMI and URBMI on health is greater than that of NRCMS, which is related to the protection levels and treatment mechanism across these systems (18). It is important to note that such disparities under system segmentation affect the realization of t universal health goals and are detrimental to social stability and harmonious development (19).

Therefore, this study's aims to: (1) compare and analyze the impact of UEBMI and URRBMI on health; (2) explore the underlying reasons for the differences between various health insurance systems; (3) evaluate the health utility of China's current social health insurance system; and (4) introduce healthcare service utilization as a mechanistic variable to further investigate the pathways of health insurance's impact on health. To provide a more comprehensive analysis, this study expands the population to include individuals aged 16 years and older, different health insurance systems from a macroscopic perspective. Additionally, this research examines how the impact of health insurance on health varies across different educational levels and regions, revealing the distinct effects of health insurance among diverse groups.

## 2 Materials and methods

### 2.1 Data and sample

Data for this study were drawn from the China Family Panel Studies (CFPS), conducted by the China Social Science Survey Center of Peking University in 2018 and 2020. The CFPS survey covers 25 provinces, municipalities, and autonomous regions across China, aiming to reflect changes in society, economy, demographics, education, and health through longitudinal data at the individual, household, and community levels. This study utilized adult and household economic questionnaires from the CFPS to collect data on individuals aged 16 years or older, with a focus on social health insurance coverage. After necessary data cleaning and merging, the final valid sample comprised 45,680 individuals.

### 2.2 Measures

#### 2.2.1 Dependent variable

Self-rated health is a comprehensive measure that integrates subjective and objective assessments of psychological, physiological, and social adaptation status. It can effectively reflect current health conditions and predict future health trends, serving as a global measure of health status for the general population (20), and holds group representativeness in China (21). In the current study, self-rated health is utilized as a measurement indicator. This research uses the self-rated health status data from the CFPS questionnaire in selecting the following question: “How do you/do you consider your health status?” The response options range from unhealthy, fair, relatively healthy, very healthy, to extremely healthy. Following previous studies, health status was dichotomized into a binary variable: responses indicating unhealthy or fair were coded as 0 (poor health), while responses of relatively healthy, very healthy, or extremely healthy are coded as 1 (good health).

#### 2.2.2 Independent variables

The independent variables in this study are URRBMI and UEBMI, selected from the questionnaire “What health insurance do you currently have?” As a reference, URRBMI was assigned a value of 1 if the respondent was enrolled in either URBMI or NRCMS, and 0 if not enrolled in any health insurance. Meanwhile, UEBMI was assigned a value of 1 if the respondent was enrolled in UEBMI, and 0 if not enrolled in any health insurance.

#### 2.2.3 Control variables

This study draws upon existing literature and employs a range of control variables, including personal characteristics, lifestyle, household characteristics, socioeconomic characteristics, and regional characteristics as the main control variables. Personal characteristics include age, gender, hukou (i.e., China's household registration system), education level, marriage, and whether or not they suffer from chronic diseases. Lifestyle refers to whether or not the respondents smoke and drink alcohol. Household characteristics are represented by household size and income per capita. Socioeconomic characteristics mainly include employment status and life satisfaction. Lastly, regional characteristics refer to the region where the survey sample belongs.

#### 2.2.4 Mechanism variables

The channel variable is healthcare utilization. It is noteworthy that the academic community mainly chooses whether or not outpatient vs. inpatient care and medical expenses occur when an illness occurs as the main measures of healthcare utilization. By combining the research topic and questionnaire data, the form of medical cost expenditure (i.e., total inpatient medical cost expenditure in the past year, outpatient medical expenditure in the past year, and out-of-pocket medical cost expenditure in the past year) and healthcare-seeking behavior (i.e., reflected by the level of healthcare facilities typically visited when ill) were used as measures of healthcare utilization.

The definition and assignment of each variable are shown in Table 1.

**Table 1:** Definition and assignment of variables.

**Variable type**	**Variable name**	**Definition**
Dependent variable	Self-rated health	1 = healthy, 0 = unhealthy
Independent variables	URRBMI	1 = insured, 0 = uninsured
	UEBMI	1 = insured, 0 = uninsured
**Control variables**		
Personal characteristics	Age	1 = young and middle-aged 16–44 years old, 2 = middle-aged 45–60 years old, 3 = middle-aged 60 years old and above
	Gender	1 = Male, 0 = Female
	Hukou	1 = agricultural, 0 = non-agricultural
	Level of education	1 = Elementary school and below, 2 = Middle and high school, 3 = College, 4 = Bachelor's degree and above
	Marriage	1 = with spouse, 0 = without spouse
	Chronic	1 = yes, 0 = no
Lifestyle	Smoke	1 = yes, 0 = no
	Drink	1 = yes, 0 = no
Household characteristics	Household income per capita	Continuous variable, log-transformed
	Household size	Continuous variable
Socio-economic characteristics	Employment status	1 = yes, 0 = no
	Life satisfaction	Continuous variable (higher scores indicate higher life satisfaction)
Region	East	1 = East, 0 = Other
	Central	1 = Central, 0 = Other
West	1 = West, 0 = Other
Mechanism variables	Medical expenses	
	Inpatient medical expenses	Continuous variable, log-transformed
	Outpatient medical expenses	Continuous variable, log-transformed
	Out-of-pocket medical expenses	Continuous variable, log-transformed
	Healthcare-seeking behavior	
	Level of healthcare facility	Higher scores indicate stronger preference for primary healthcare institutions

### 2.3 Model settings

#### 2.3.1 Logit model

The dependent variable is self-rated health, represented by a dichotomous dummy variable with values of 0 and 1. The independent variables include URRBMI and UEBMI, both similarly structured as binary dummy variables. Therefore, a logit model was selected for this analysis, which can be described as follows.


(1) $$\begin{array}{l} In\left(\frac{P_{i}}{1-P_{i}}\right)=\alpha +\beta_{0}X_{i}+\sum \beta_{j}Z_{ij}, \end{array}$$

where *i* represents the *i*th individual, *P*_*i*_ denotes the probability of self-rated health, *X*_*i*_ stands for social health insurance, and *Z*_*ij*_ signifies the control variables.

#### 2.3.2 Propensity score matching method

The independent variable constitutes a dichotomous selection variable. However, the inclusion of URRBMI does not strictly follow the exogenous event occurring at random, participation in insurance is influenced by individual characteristics, economic status, and social resources, among other factors. This influence introduces a degree of selection bias into the sample. To address this issue, the present study employs propensity score matching (PSM) methodology. The model specification proceeds as follows.


(2) $$\begin{array}{l} {\text{Y}}_{\text{i}}={\text{Y}}_{0\text{i}}+\left({\text{Y}}_{1\text{i}}-{\text{Y}}_{0\text{i}}\right){\text{D}}_{\text{i}}, \end{array}$$

(3) $$\begin{array}{l} \text{ATT}=\text{E}\left({\text{Y}}_{1\text{i}}-{\text{Y}}_{0\text{i}}|{\text{D}}_{\text{i}}=1\right), \end{array}$$

where *D*_*i*_ is the treatment variable: when *i* equals 1, individual *i* belongs to the experimental group (i.e., individuals who participated in URRBMI), and when *i* equals 0, individual *i* belongs to the control group (i.e., individuals who indicated they did not participate in URRBMI); and ATT (i.e., average treatment effect on the treated) represents the net effect of URRBMI on the self-rated health of insured individuals.

#### 2.3.3 Instrumental variable method

This study used PSM to address the sample selection bias of URRBMI. Hence, the focus is on the endogeneity problem of UEBMI, which is addressed by the instrumental variable (IV) method. The current research uses the introduction of instrumental variable as a foundational element in analyzing the impact of health insurance on self-rated health using the two-stage least squares (2SLS) method. In the empirical analysis employing 2SLS, the relationship between UEBMI and the instrumental variable must be estimated first. That is the first stage estimation, which takes the following form, constitutes the first step:


(4) $$\begin{array}{l} {\text{X}}_{\text{insurance}}=\beta_{1}+\beta_{2}\text{I}\text{V}+\overset{\text{j}}{\underset{1}{\sum }}\beta_{\text{j}}{\text{control}}_{\text{j}}+\varepsilon . \end{array}$$

the second stage is estimated with the following regression model:


(5) $$\begin{array}{l} {\text{Y}}_{\text{health}}=\gamma_{1}+\gamma_{2}{\text{X}}_{\text{insurance}}+\overset{\text{j}}{\underset{1}{\sum }}\beta_{\text{j}}{\text{control}}_{\text{j}}+\varepsilon . \end{array}$$

in Equation 1, *X*_*insurance*_ is the independent variable of whether or not to participate in UEBMI and IV is the health insurance instrumental variable (i.e., respondent's job type). In Equation 2, *Y*_*health*_ is the dependent variable self-rated health status, *control*_*j*_ is the control variable, and ε is the residual term.

## 3 Results

### 3.1 Descriptive statistical analysis

According to the descriptive statistics in Table 2, the age distribution of the sample is mainly concentrated around 45 years old, with a nearly equal gender ratio. The majority of the participants originate from agricultural backgrounds and exhibit generally low levels of education. Most individuals rate their health as relatively good, with 71.27% reporting no smoking habits in daily life, and 86% indicating no alcohol consumption on a regular basis. Only 16.59% of the sample reports suffering from chronic diseases. Participation rates in UEBMI and URRBMI are 63.18% and 89.09%, respectively. Household sizes for most respondents average four persons, consistent with the size of an average household. The per capita household income for the majority of the sample falls within the middle range. Additionally, most of the samples come from the eastern region. Fewer than half of the sample incurred hospitalization expenses in the past year, while a larger proportion reported outpatient medical expenditures. Lastly, respondents showed a preference for primary care when seeking medical treatment.

Table 2Descriptive statistical analysis.

**Table:** 

**Panel A: categorical variables**			
**Variable**	**Category**	**Frequency**	**Percentage (%)**
Self-rated health	1 = healthy	32,360	70.84
	0 = unhealthy	13,320	29.16
URRBMI	1 = insured	31,547	89.09
	0 = uninsured	3,863	10.91
UEBMI	1 = insured	6,628	63.18
	0 = uninsured	3,863	36.82
Age	1 = young and middle-aged 16–44 years old	19,929	43.63
	2 = middle-aged 45–60 years old,	13,940	30.52
	3 = middle-aged 60 years old and above	11,811	25.86
Gender	1 = Male	22,695	49.68
	0 = Female	22,985	50.32
Hukou	1 = agricultural	33,672	79.88
	0 = non-agricultural	8,479	20.12
Level of education	1 = Elementary school and below	31,341	68.61
	2 = Middle and high school	10,904	23.87
	3 = College	1,513	3.31
	4 = Bachelor's degree and above	1,922	4.21
Marriage	1 = with spouse	36,009	79.61
	0 = without spouse	9,225	20.39
Chronic	1 = yes	6,991	16.59
	0 = no	35,160	83.41
Smoke	1 = yes	12,112	28.73
	0 = no	30,039	71.27
Drink	1 = yes	6,046	14.34
	0 = no	36,104	85.66
Employment status	1 = yes	33,342	78.02
	0 = no	9,392	21.98
East	1 = East	18,390	40.28
	0 = Other	27,266	59.72
Central	1 = Central	13,402	29.35
	0 = Other	32,254	70.65
West	1 = West	13,864	30.37
	0 = Other	31,792	69.63

**Table:** 

**Panel B: continuous variables**					
**Variable**	***N***	**Mean**	**Std. Dev**.	**Min**	**Max**
Household income per capita	45,680	9.711	0.959	6.891	12.346
Household size	45,680	4.135	1.877	1	10
Life satisfaction	42,151	4.017	0.946	1	5
Inpatient medical expenses	39,513	8.824	1.275	0	11.918
Outpatient medical expenses	43,530	2.446	3.675	0	9.904
Out-of-pocket medical expenses	28,124	6.56	1.954	0	10.645
Level of healthcare facility	42,151	3.143	1.481	1	5

### 3.2 Benchmark regression

Table 3 indicates that models (1)–(3) asymptotically show the relationships among full sample health insurance, URRBMI, UEBMI, and self-rated health. Model (1) shows that when full-sample health insurance is used as the independent variable to examine its overall effect on self-rated health, the impact of social health insurance on health is positive but statistically insignificant, potentially due to the combined effects of different types of health insurance. Models (2) and (3) report the respective effects of URRBMI and UEBMI, respectively, on self-rated health, showing that the coefficient for URRBMI's effect on self-rated health is positive but non-significant. This finding suggests that URRBMI does not significantly improve participants' self-rated health. In contrast, the coefficient for UEBMI on self-rated health is both positive and significant, indicating that UEBMI effectively improves enrollees' self-rated health. Furthermore, comparison with the uninsured group shows that UEBMI's utility in improving self-rated health is better than that of URRBMI.

**Table 3:** Benchmark regression results.

**Variable**	**(1)**	**(2)**	**(3)**	**(4)**
	**Self-rated health**	**Self-rated health**	**Self-rated health**	**Self-rated health**
Full sample health insurance	0.070			
	(1.58)			
URRBMI		0.053		
		(1.17)		
UEBMI			0.248^***^	0.109^**^
			(3.89)	(2.52)
Age	−0.533^***^	−0.543^***^	−0.571^***^	−0.512^***^
	(−30.85)	(−29.55)	(−14.22)	(−28.29)
Gender	0.234^***^	0.234^***^	0.182^***^	0.227^***^
	(7.54)	(6.91)	(2.85)	(6.97)
Hukou	−0.107^***^	−0.038	−0.047	−0.090^**^
	(−3.21)	(−0.92)	(−0.79)	(−2.36)
Level of education	0.406^***^	0.488^***^	0.246^***^	0.395^***^
	(17.49)	(16.62)	(6.88)	(15.87)
Marriage	−0.224^***^	−0.202^***^	−0.360^***^	−0.205^***^
	(−6.27)	(−5.22)	(−5.11)	(−5.36)
Chronic	−1.344^***^	−1.349^***^	−1.319^***^	−1.348^***^
	(−45.16)	(−41.66)	(−20.87)	(−43.64)
Smoke	0.070^**^	0.088^**^	−0.062	0.091^***^
	(2.07)	(2.40)	(−0.89)	(2.58)
Drink	0.134^***^	0.120^***^	0.227^***^	0.130^***^
	(3.58)	(2.98)	(2.81)	(3.31)
Household income per capita	0.203^***^	0.191^***^	0.185^***^	0.200^***^
	(14.32)	(12.66)	(5.42)	(13.15)
Household size	0.023^***^	0.025^***^	0.022	0.023^***^
	(3.35)	(3.45)	(1.36)	(3.27)
Employment status	0.376^***^	0.398^***^	0.337^***^	0.379^***^
	(12.77)	(12.52)	(5.16)	(12.21)
Life satisfaction	0.328^***^	0.330^***^	0.323^***^	0.329^***^
	(25.93)	(24.62)	(11.64)	(24.64)
East	0.046	0.059^*^	−0.103	0.054^*^
	(1.53)	(1.81)	(−1.42)	(1.71)
Central	0.091^***^	0.100^***^	−0.008	0.089^***^
	(2.88)	(2.97)	(−0.10)	(2.70)
_cons	−1.978^***^	−2.059^***^	−1.261^***^	−1.965^***^
	(−11.61)	(−11.34)	(−3.27)	(−11.02)
*N*	40,486.000	33,869.000	10,091.000	37,012.000

Standard errors in parentheses: ^*^p < 0.1, ^**^p < 0.05, and ^***^p < 0.01.

To further validate the above conclusions, we focused on the insured sample in Model (4) by assigning values of 0 and 1 to the enrollee's participation in URRBMI and UEBMI, respectively. By directly comparing the health effects of the two systems among the insured, we aimed to more accurately assess their true impact on health outcomes and compare their relative effects after enrollment. The results show that, compared to URRBMI, UEBMI has a statistically significant positive effect on self-rated health, further supporting the findings of Model (3), that it is more effective in safeguarding and improving health.

For personal characteristics, age has a significant negative effect on self-rated health, indicating that younger individuals report better self-rated health compared to older adults. Gender has a significant positive effect on self-rated health, with women reporting worse self-rated health than men, potentially due to the additional burdens of marriage and household labor. The results also indicate that marital status exerts a negative influence on self-rated health. Hukou type does not show a significant effect on self Meanwhile, education level has a significant positive effect on self-rated health, likely because higher education level is usually associated with higher income and greater access to healthcare resources. For household characteristics, income has a positive effect on health, as higher-income groups can leverage their financial resources to improve living conditions. Household size generally has a positive effect on self-rated health, with daily care and emotional support from family members contributing to both physical and mental wellbeing. Employment status has a significant positive effect on self-rated health, possibly due to the psychological benefits derived from a sense of achievement at work. Life satisfaction has a significant positive effect on self-rated health, suggesting that considerably high quality of life and subjective wellbeing contribute to physical and mental health.

### 3.3 Endogenous treatment

#### 3.3.1 PSM processing

Given that URRBMI is a non-mandatory enrollment scheme, individual differences in selectivity may lead to sample selection bias. To mitigate this bias, this study uses PSM for estimation.

Table 4 presents the changes in the sample before and after matching. The results show that the standard deviation of each variable is significantly reduced after matching, with values below 10%. Additionally, all t-test results after matching accept the original hypothesis, indicating no systematic difference between the treatment and control groups. These findings validate the effectiveness and reliability of the matching procedure.

**Table 4:** Changes in sample variables before and after PSM.

**Variable**	**Unmatched**	**Mean**		**%bias**	**%reduct |bias**	***T*** **-test**		**V(T)/V(C)**
	**Matched**	**Treated**	**Control**			***t***	***p*** > ***t***	
Age	U	1.885	1.708	21.9	.	12.37	0.000	0.94^*^
	M	1.885	1.920	−4.4	79.9	−5.37	0.000	0.90^*^
Gender	U	0.486	0.487	−0.1		−0.07	0.941	.
	M	0.486	0.457	5.9	−4,359.1	7.26	0.000	.
Hukou	U	0.885	0.763	32.6	.	20.64	0.000	.
	M	0.885	0.881	1.0	96.9	1.45	0.146	.
Level of education	U	1.291	1.479	−28.2	.	−18.05	0.000	0.54^*^
	M	1.291	1.281	1.4	94.9	2.08	0.038	0.94^*^
Marriage	U	0.861	0.705	38.6	.	24.30	0.000	.
	M	0.861	0.867	−1.4	96.3	−2.11	0.035	.
Chronic	U	0.176	0.139	10.2	.	5.50	0.000	.
	M	0.176	0.166	2.6	74.2	3.14	0.002	.
Smoke	U	0.299	0.302	−0.5	.	−0.29	0.772	.
	M	0.299	0.282	3.8	−627.6	4.70	0.000	.
Drink	U	0.152	0.142	2.6	.	1.43	0.153	.
	M	0.152	0.151	0.3	90.3	0.31	0.760	.
Household income per capita	U	9.568	9.768	−20.8	.	−12.12	0.000	0.81^*^
	M	9.568	9.535	3.3	84.0	4.14	0.000	0.83^*^
Household size	U	4.243	3.806	22.7	.	12.85	0.000	0.94^*^
	M	4.243	4.255	−0.6	97.3	−0.75	0.451	0.90^*^
Employment status	U	0.782	0.709	16.8	.	9.77	0.000	.
	M	0.782	0.763	4.3	74.4	5.50	0.000	.
Life satisfaction	U	4.044	3.887	15.5	.	9.01	0.000	0.82^*^
	M	4.044	4.043	0.1	99.4	0.11	0.912	0.88^*^
East	U	0.371	0.530	−32.4	.	−18.34	0.000	.
	M	0.371	0.385	−2.8	91.4	−3.50	0.000	.
Central	U	0.295	0.277	4.0	.	2.20	0.028	.
	M	0.295	0.287	2.0	50.5	2.41	0.016	.
West	U	0.334	0.192	32.5	.	16.99	0.000	.
	M	0.334	0.329	1.1	96.6	1.27	0.203	.

Standard errors in parentheses: ^*^p < 0.1.

Table 5 shows the ATT for URRBMI. The absolute value of the matched *t*-test is 1.14, which is below the critical value of 1.67, and the coefficient is positive. This result indicates that URRBMI has a positive but non-significant effect on health, consistent with the results of the baseline regression.

**Table 5:** URRBMI PSM mean treatment effects.

**Variable**	**Matching**	**Treated**	**Control**	**Difference**	**Standard error**	**T-statistic**
Self-rated health	Before	0.689093601	0.714450201	−0.025356601	0.00826962	−3.07
	ATT	0.689095815	0.674453804	0.014642011	0.12290141	1.19
	ATU	0.714367982	0.723082829	0.008714848		
	ATE			0.014034155		

#### 3.3.2 IV processing

UEBMI does not face the same situation as URRBMI, where enrollment is mandatory rather than based on voluntary choice. Therefore, sample selection bias is not the primary endogeneity issue confronting UEBMI. Instead, omitted variables or reverse causality may pose challenges. To address these endogeneity concerns, this study employs IV methodology. Specifically, following Shao et al. (22), job type is selected as the IV. The CFPS categorizes job types into five main categories: own-family agricultural production and operation, private enterprise/individually owned business/other self-employment, part-time agricultural work, employed, and non-agricultural casual labor. For relevance, individuals engaged in private enterprise/individually owned business/other self-employed and employed are more likely to be enrolled in social health insurance, predominantly UEBMI. Conversely, those involved in home-based agricultural production operations, agricultural part-time jobs, and non-farm casual laborers are less likely to be insured due to the absence of employer-mandated enrollment. Thus, a Thus, a significant correlation exists between employment type and social health insurance enrollment. For exogeneity, after controlling for relevant variables strongly linked to self-rated health, job type does not directly influence the level of self-rated health. Therefore, the selected IV theoretically satisfies the requirements for instrumental variable.

As shown in Table 6, the results of the Durbin-Wu-Hausman test indicate the rejection of the original hypothesis, suggesting a serious two-way causality problem between the two core variables. Therefore, IV must be introduced for robustness testing. The correlation test indicated that this IV is not weak (F > 10), its validity has been confirmed in practice. After introducing the IV, the first-stage regression results are significantly positive. Furthermore, the second-stage regression results demonstrate that the effect of UEBMI on self-rated health remains significantly positive. This finding is consistent with the benchmark regression results, indicating the reliability of the benchmark regression findings.

**Table 6:** UEBMI endogenous treatment.

**Variable**	**(1)**	**(2)**
	**First stage**	**Second stage**
UEBMI		0.173^***^
		(4.97)
Age	0.050^***^	−0.091^***^
	(6.30)	(−12.77)
Gender	0.045^***^	0.015
	(4.19)	(1.46)
Hukou	−0.208^***^	0.033^***^
	(−22.34)	(2.71)
Level of education	0.063^***^	0.011^**^
	(13.21)	(2.20)
Marriage	0.133^***^	−0.068^***^
	(11.87)	(−5.90)
Chronic	0.040^***^	−0.274^***^
	(2.90)	(−21.12)
Smoke	−0.042^***^	−0.003
	(−3.66)	(−0.26)
Drink	0.003	0.027^**^
	(0.23)	(2.17)
Household income per capita	0.120^***^	0.008
	(20.26)	(1.01)
Household size	0.005^*^	0.001
	(1.68)	(0.33)
Employment status	0.120^***^	0.024^*^
	(8.33)	(1.77)
Life satisfaction	0.023^***^	0.050^***^
	(4.63)	(10.84)
East	−0.048^***^	−0.007
	(−4.04)	(−0.64)
Central	−0.034^***^	0.012
	(−2.64)	(0.95)
Work type (IV)	0.130^***^	
	(27.17)	
_cons	−1.412^***^	0.562^***^
	(−20.76)	(7.28)
*N*	8,053	8,053
r2	0.336	0.122
Cragg-Donald Wald F statistic	738.037	
Durbin-Wu-Hausman Score Test		
χ^2^statistic		14.54
p-value		0.0001

Standard errors in parentheses: ^*^p < 0.1, ^**^p < 0.05, and ^***^p < 0.01.

### 3.4 Heterogeneity analysis

#### 3.4.1 Heterogeneity analysis of educational level

Studies have shown that education has a significant positive effect on self-rated health. Individuals with higher levels of education are more adept at acquiring and applying complex treatment plans, thereby managing their diseases more effectively (23). Conversely, individuals with lower levels of education have lower rates of acceptance and translation of health knowledge and fewer preventive health behaviors owing to insufficient health literacy, resulting in higher hospitalization rates and lower levels of self-rated health (24). Therefore, this study investigates the heterogeneity of self-rated health across different education levels. The regression results are shown in Table 7. Notably, regardless of education level, UEBMI consistently exhibits a stronger positive effect on self-rated health compared to URRBMI. This improvement is particularly pronounced among individuals with primary education. Furthermore, the positive effect of UEBMI on self-rated health increases with education level, although it is no longer significant as education level increases.

**Table 7:** Heterogeneity in educational level.

**Variable**	**Elementary school**		**Secondary school**		**College**		**Bachelor's degree and above**	
	**(1)**	**(2)**	**(3)**	**(4)**	**(5)**	**(6)**	**(7)**	**(8)**
URRBMI	0.064		−0.028		0.115		0.198	
	(1.25)		(−0.28)		(0.32)		(0.58)	
UEBMI		0.333^***^		0.089		−0.189		0.388
		(4.10)		(0.70)		(−0.55)		(1.19)
Age	−0.513^***^	−0.527^***^	−0.643^***^	−0.645^***^	−0.631^**^	−0.262	−0.258	−0.791^***^
	(−25.44)	(−10.94)	(−13.66)	(−7.62)	(−2.36)	(−1.14)	(−0.70)	(−2.86)
Gender	0.239^***^	0.133^*^	0.185^**^	0.200	0.349	0.351	0.620^*^	0.279
	(6.37)	(1.65)	(2.19)	(1.55)	(1.03)	(1.18)	(1.65)	(1.08)
Hukou	−0.087^*^	−0.040	0.151	−0.052	0.368	0.307	−0.105	−0.156
	(−1.87)	(−0.50)	(1.56)	(−0.48)	(1.16)	(1.30)	(−0.27)	(−0.68)
Marriage	−0.181^***^	−0.285^***^	−0.262^***^	−0.504^***^	−0.517	−0.373	−0.574	−0.481
	(−4.17)	(−3.21)	(−2.67)	(−3.44)	(−1.58)	(−1.24)	(−1.52)	(−1.57)
Chronic	−1.292^***^	−1.228^***^	−1.572^***^	−1.522^***^	−2.065^***^	−1.264^***^	−0.789	−1.337^***^
	(−36.31)	(−15.40)	(−19.42)	(−12.52)	(−6.27)	(−4.39)	(−1.60)	(−4.72)
Smoke	0.103^**^	−0.063	0.009	0.038	0.424	−0.186	−0.540	−0.566^*^
	(2.54)	(−0.72)	(0.10)	(0.27)	(1.00)	(−0.58)	(−1.15)	(−1.85)
Drink	0.139^***^	0.212^**^	0.059	0.410^**^	−0.377	−0.252	−0.615	−0.065
	(3.16)	(2.17)	(0.60)	(2.38)	(−0.74)	(−0.67)	(−1.07)	(−0.15)
Household income per capita	0.202^***^	0.222^***^	0.167^***^	0.082	0.062	−0.010	−0.363	0.030
	(12.21)	(5.38)	(4.30)	(1.09)	(0.37)	(−0.06)	(−1.64)	(0.17)
Household size	0.021^***^	0.020	0.038^**^	0.015	−0.010	0.010	0.052	−0.007
	(2.59)	(1.05)	(2.04)	(0.46)	(−0.13)	(0.13)	(0.46)	(−0.09)
Employment status	0.421^***^	0.430^***^	0.360^***^	0.270^*^	0.144	0.162	0.215	−0.357
	(11.91)	(5.44)	(4.59)	(1.95)	(0.42)	(0.44)	(0.57)	(−0.67)
	0.312^***^	0.297^***^	0.399^***^	0.369^***^	0.702^***^	0.498^***^	0.718^***^	0.516^***^
Life satisfaction	(21.22)	(8.84)	(11.69)	(6.33)	(4.52)	(3.67)	(3.44)	(3.26)
	0.059^*^	−0.139	0.026	−0.121	0.179	0.110	−0.300	0.010
East	(1.65)	(−1.52)	(0.32)	(−0.80)	(0.55)	(0.37)	(−0.65)	(0.03)
	0.082^**^	−0.038	0.137	−0.122	0.479	0.383	−0.304	0.232
Central	(2.22)	(−0.38)	(1.60)	(−0.75)	(1.30)	(1.21)	(−0.61)	(0.71)
	−1.682^***^	−1.540^***^	−0.842^*^	0.681	−0.319	0.651	3.778	1.434
_cons	(−8.46)	(−3.23)	(−1.86)	(0.80)	(−0.17)	(0.34)	(1.47)	(0.69)
	0.064		−0.028		0.115		0.198	
*N*	25,053	5,254	7,566	2,964	797	851	453	1,022

Standard errors in parentheses: ^*^p < 0.1, ^**^p < 0.05, and ^***^p < 0.01.

#### 3.4.2 Regional heterogeneity analysis

Analysis revealed that social health insurance has significant health performance and regional heterogeneity at the geographic location level, closely related to variations in the level and efficiency of health services between regions (25). Therefore, this study further analyzed the impact of social health insurance on self-rated health across different regions. The results are shown in Table 8. Notably, both URRBMI and UEBMI have a positive and significant effect on the self-rated health of enrollees in the eastern region. indicating that both types of enrollments can enhance residents' self-rated health in this area. However, URRBMI has a negative but non-significant effect on self-rated health. In contrast, UEBMI has a negative and insignificant effect on self-rated health in the central and western regions, suggesting that while it may have some positive impact, its overall effect is limited. Overall, URRBMI demonstrates a weaker effect on self-rated health compared to UEBMI across all regions.

**Table 8:** Heterogeneity in regional.

**Variable**	**Eastern region**		**Central region**		**Western region**	
	**(1)**	**(2)**	**(3)**	**(4)**	**(5)**	**(6)**
URRBMI	0.126^**^		−0.030		−0.058	
	(2.01)		(−0.34)		(−0.59)	
UEBMI		0.312^***^		0.126		0.168
		(3.66)		(1.04)		(1.01)
Age	−0.605^***^	−0.603^***^	−0.473^***^	−0.413^***^	−0.531^***^	−0.728^***^
	(−20.08)	(−10.74)	(−13.65)	(−5.58)	(−16.74)	(−7.67)
Gender	0.271^***^	0.335^***^	0.175^***^	0.124	0.216^***^	−0.206
	(5.05)	(3.78)	(2.81)	(1.06)	(3.50)	(−1.31)
Hukou	0.051	0.057	−0.098	−0.146	−0.194^**^	−0.298^**^
	(0.84)	(0.71)	(−1.35)	(−1.35)	(−2.04)	(−1.98)
Level of education	0.405^***^	0.230^***^	0.574^***^	0.322^***^	0.509^***^	0.183^**^
	(9.08)	(4.62)	(10.44)	(4.88)	(9.12)	(2.17)
Marriage	−0.213^***^	−0.304^***^	−0.045	−0.340^***^	−0.316^***^	−0.582^***^
	(−3.40)	(−3.12)	(−0.62)	(−2.64)	(−4.69)	(−3.37)
Chronic	−1.331^***^	−1.295^***^	−1.355^***^	−1.372^***^	−1.372^***^	−1.295^***^
	(−24.75)	(−14.72)	(−22.85)	(−12.06)	(−24.44)	(−8.39)
Smoke	0.063	−0.129	0.038	−0.091	0.180^***^	0.209
	(1.08)	(−1.32)	(0.56)	(−0.72)	(2.73)	(1.25)
Drink	0.220^***^	0.254^**^	0.142^*^	0.165	−0.039	0.199
	(3.42)	(2.29)	(1.91)	(1.11)	(−0.53)	(0.96)
Household income per capita	0.202^***^	0.167^***^	0.198^***^	0.196^***^	0.173^***^	0.212^**^
	(8.58)	(3.64)	(6.82)	(3.03)	(6.42)	(2.51)
Household size	0.015	0.002	0.037^***^	0.062^**^	0.026^**^	0.007
	(1.31)	(0.11)	(2.78)	(2.09)	(2.05)	(0.20)
Employment status	0.416^***^	0.316^***^	0.481^***^	0.462^***^	0.270^***^	0.139
	(8.46)	(3.46)	(8.38)	(3.94)	(4.39)	(0.88)
Life satisfaction	0.393^***^	0.374^***^	0.336^***^	0.266^***^	0.253^***^	0.287^***^
	(17.94)	(9.75)	(13.44)	(5.25)	(10.89)	(4.21)
_cons	−2.267^***^	−1.423^***^	−2.385^***^	−1.639^**^	−1.186^***^	−0.297
	(−7.79)	(−2.71)	(−6.99)	(−2.30)	(−3.57)	(−0.31)
*N*	13,119	5,330	9,944	2,955	10,806	1,806

Standard errors in parentheses: ^*^p < 0.1, ^**^p < 0.05, and ^***^p < 0.01.

### 3.5 Mechanism analysis

This study examines the mechanism of action concerning healthcare-seeking behavior and medical service consumption. Variables related to medical consumption were selected to represent hospitalization costs, non-hospitalization costs, and out-of-pocket expenses incurred over the past year. Variables pertaining to medical choice were chosen to reflect the types of medical institutions that insured individuals prefer to visit. The results are shown in Table 9. The analysis is as follows.

**Table 9:** Mechanism of action regression results.

**Variable**	**(1)**	**(2)**	**(3)**	**(4)**
UEBMI	0.966^***^	0.190^***^	−0.455^*^	0.144
	(0.342)	(0.072)	(0.255)	(0.094)
Inpatient medical expenses	−0.121^***^			
	(0.031)			
Outpatient medical expenses		−1.510^***^		
		(0.148)		
Out-of-pocket medical expenses			−0.258^***^	
			(0.030)	
Level of healthcare facility				0.091^***^
				(0.026)
Age	−0.564^***^	−0.530^***^	−0.553^***^	−0.570^***^
	(0.044)	(0.041)	(0.049)	(0.040)
Gender	0.183^***^	0.185^***^	0.100	0.183^***^
	(0.069)	(0.066)	(0.076)	(0.064)
Hukou	−0.053	−0.040	−0.045	−0.065
	(0.064)	(0.061)	(0.072)	(0.059)
Level of education	0.253^***^	0.246^***^	0.207^***^	0.254^***^
	(0.039)	(0.037)	(0.042)	(0.036)
Marriage	−0.238^***^	−0.376^***^	−0.306^***^	−0.354^***^
	(0.076)	(0.073)	(0.084)	(0.070)
Chronic	−1.211^***^	−1.048^***^	−0.906^***^	−1.298^***^
	(0.067)	(0.067)	(0.072)	(0.063)
Smoke	−0.025	−0.089	−0.044	−0.067
	(0.076)	(0.072)	(0.084)	(0.070)
Drink	0.269^***^	0.193^**^	0.111	0.223^***^
	(0.089)	(0.083)	(0.099)	(0.081)
Household income per capita	0.214^***^	0.212^***^	0.206^***^	0.202^***^
	(0.038)	(0.035)	(0.042)	(0.034)
Household size	0.038^**^	0.020	0.008	0.019
	(0.017)	(0.016)	(0.019)	(0.016)
Employment status	0.256^***^	0.232^***^	0.212^***^	0.320^***^
	(0.072)	(0.068)	(0.081)	(0.066)
Life satisfaction	0.317^***^	0.317^***^	0.248^***^	0.326^***^
	(0.030)	(0.029)	(0.034)	(0.028)
East	−0.149^*^	−0.095	−0.262^***^	−0.096
	(0.078)	(0.075)	(0.085)	(0.073)
Central	−0.014	−0.038	−0.102	−0.008
	(0.085)	(0.081)	(0.092)	(0.079)
UEBMI ^*^ inpatient medical expenses	−0.084^**^			
	(0.042)			
UEBMI ^*^ outpatient medical expenses		0.354^**^		
		(0.173)		
UEBMI ^*^ out-of-pocket medical expenses			0.102^***^	
			(0.035)	
UEBMI ^*^ level of healthcare facility				0.057^*^
				(0.030)
_cons	−0.778	−1.280^***^	0.475	−1.338^***^
	(0.475)	(0.398)	(0.514)	(0.386)
*N*	8,404	9,978	6,118	10,091

Standard errors in parentheses: ^*^p < 0.1, ^**^p < 0.05, and ^***^p < 0.01.

#### 3.5.1 Mechanism of hospitalization consumption

According to model (1), UEBMI significantly enhances the self-rated health of enrollees by reducing the cost of inpatient medical care. This is attributed to UEBMI enrollees having relatively higher income levels, access to high-quality medical services, and a reduced impact on self-rated health, leading to a markedly lower demand for hospitalization. Moreover, the dual structure between urban and rural areas has led to greater availability of medical services in urban China, thereby lowering the risk of minor illnesses progressing into major ones. The urban-rural dual structure ensures high accessibility of medical services in urban regions, reducing the likelihood of “minor illnesses” being delayed and becoming “major illnesses” (26). Additionally, regular health checkups for UEBMI participants facilitate early detection and treatment of illnesses, further decreasing the need for hospitalization. Primary care and outpatient services play a substitution role for inpatient care (27), and the outpatient protection system under UEBMI effectively reduces the utilization rate of inpatient services. Following the implementation of outpatient coordination under UEBMI, inpatient costs have significantly decreased, while primary care visits and outpatient service utilization have increased, thereby fulfilling a substitution role for inpatient services (28).

#### 3.5.2 Mechanism of the role of outpatient consumption

Model (2) reveals that the coefficient of the interaction term between health insurance and outpatient costs is significantly positive. This finding indicates that UEBMI is more effective in enhancing self-rated health by promoting the utilization of outpatient care services compared to URRBMI. Furthermore, UEBMI encourages enrollees to utilize outpatient care services due to the availability of a personal account and higher reimbursement rates for both outpatient and inpatient care, as confirmed by existing research (12). Additionally, UEBMI operates as a current income account, which presents a strong consumption incentive; the higher the account balance, the greater the incentive to consume (29). Consequently, as account funds accumulate, participants are more likely to increase their utilization of outpatient healthcare services, such as outpatient consultations (30).

#### 3.5.3 Mechanism of self-paid medical consumption

According to model (3), the coefficient of the interaction term between UEBMI and self-paid medical consumption is significantly positive. This finding indicates that UEBMI enhances the level of self-rated health by increasing self-paid medical consumption, thereby improving the health status of enrollees. Previous analysis has demonstrated that UEBMI increases enrollees' utilization of medical services; an increase in healthcare utilization is typically accompanied by a rise in self-paid medical expenses. Consequently, compared to the uninsured, UEBMI participants utilize outpatient and inpatient medical services more frequently, resulting in higher actual self-paid medical consumption. Increased healthcare utilization enables insured patients to identify and address their health issues more promptly. Therefore, the underlying cause of increased self-paid medical consumption under UEBMI can be attributed to enhanced healthcare utilization.

#### 3.5.4 Mechanism of medical care choice

Model (4) shows that the coefficient of the interaction term between UEBMI and visiting medical institutions is significantly positive, indicating that UEBMI has a stronger positive effect on self-rated health among individuals who are more likely to utilize primary healthcare services, reflecting the effectiveness of China's current tiered healthcare delivery system. Higher reimbursement rates and lower deductibles for primary care, along with the inclusion of general outpatient expenses in the scope of the overall fund payment, reducing the out-of-pocket expenses of insured persons for primary healthcare treatment, Additionally, many regions require UEBMI beneficiaries to select primary medical institutions as the first diagnosis institutions otherwise they cannot enjoy high reimbursement rates. These reforms encourage primary care utilization for common and chronic conditions, mitigating financial barriers and improving health outcomes.

## 4 Discussion

### 4.1 Conclusion

This study used data from the CFPS in 2018 and 2020 as bases to explore the relationship between URRBMI and UEBMI and self-rated by using logit model, PSM method, and IV method. The regression results show differences in the effects of different types of health insurance systems on enrollees' self-rated health. Specifically, UEBMI demonstrates a more pronounced positive impact on improving enrollees' self-rated health compared to URRBMI. The key conclusions are as follows:

The positive effect of URRBMI on participants' self-rated health is not significant. The reasons are as follows. First, URRBMI lacks the dual protection of coordinated and individual accounts. Second, the level of reimbursement for inpatient medical services is lower than that of urban workers. Lastly, the protection effect on outpatient care is not as strong, thereby inhibiting the healthcare utilization by URRBMI participants and leading to deterioration in health. In addition, the integration of URRBMI has led to inconsistencies in institutional policy and administration across the region and differences in the modes of operation and service. These results, coupled with the disparities between urban and rural areas in the allocation of medical resources, economic levels, health protection, and concepts of medical care, have made the impact of URRBMI on self-rated health not observable.

UEBMI has a significant positive impact on self-rated health. The dual structure of individual and pooled accounts within UEBMI facilitates greater healthcare utilization, thereby enhancing self-rated health among participants. Furthermore, these participants reside in urban areas, where they have access to high-quality medical resources and services. Additionally, workplaces of UEBMI participants often organize regular health check-ups, which aid in the early detection and prevention of illnesses, thus safeguarding their overall health.

UEBMI enhances participants' self-rated health through three primary mechanisms: reducing inpatient healthcare consumption, increasing outpatient healthcare utilization, and promoting the use of large hospitals' healthcare services. The mechanisms are as follows. First, high-income participants have a strong sense of health management, leading to better overall health and a reduced need for hospitalization. Consequently, they incur lower hospitalization costs. The higher outpatient coverage by the UEBMI individual account promotes the use of outpatient services, which helps prevent minor illnesses from becoming serious, thus reducing the need for inpatient care. Second, UEBMI improves participants' self-rated health by enhancing the consumption of non-inpatient care. Lastly, UEBMI guides participants to prioritize primary healthcare institutions for the management of common and chronic diseases to improve the level of self-rated health.

Heterogeneity exists in the effects of URRBMI and urban workers' health insurance on self-rated health. Specifically, the impact of different types of social health insurance on self-rated health varies by educational attainment and geographic region. Overall, UEBMI demonstrates superior outcomes compared to URRBMI, particularly with respect to educational level and regional disparities. This advantage is primarily attributed to UEBMI's dual protection mechanism, which includes both pooled and individual accounts. This dual structure effectively distributes the financial burden of inpatient and outpatient care, enhances healthcare utilization rates, and ultimately improves the insured's self-rated health.

### 4.2 Policy implications

#### 4.2.1 Improve the design of medical insurance system

China's UEBMI has long outperformed URRBMI in outpatient and inpatient benefits. This “binary division” undermines the fairness and sustainability of the system. To address this, URRBMI financing should consider local economic levels, financial strength, and residents' income, adjust the growth and reimbursement rates according to social development, and unify payment standards for medical services. Additionally, enterprises and other departments should be encouraged to provide supplementary subsidies to increase insurance coverage and enhance protection for minor illnesses, thus preventing escalation to major illnesses. Finally, medical examination expenses should be included in the reimbursement scope of URRBMI. Self-payment for these expenses discourages proactive health check-ups, which is detrimental to maintaining health from the outset.

#### 4.2.2 Multi-channel to narrow the income gap between residents

Income has a positive effect on health, as higher income facilitates better access to social resources and promotes overall wellbeing. Improved health, in turn, enhances labor productivity and income, forming a virtuous cycle. The government should leverage China's common prosperity initiative to narrow the income gap by improving the income distribution system and enhancing the income levels and stability of low- and middle-income groups. This can be achieved through the development of local industries, promotion of entrepreneurship and employment, increases in the minimum wage, and strengthened policy support for these groups. Additionally, the government should enhance financial transfers, boost national investment in the social medical insurance system in central and western regions, improve the resilience of the medical insurance fund, and strengthen health protection for residents in these areas.

#### 4.2.3 Deepen the collaborative reform of medical insurance at the grass-roots level

To sustain the positive impact of employee medical insurance on self-rated health, policies should further promote the tiered diagnosis and treatment system, expand reimbursement coverage and ratios at primary care institutions, and guide residents to seek care at the appropriate level. This will help improve health outcomes and establish a virtuous cycle between medical insurance and primary care development. On the institutional side, efforts should focus on optimizing resource allocation, strengthening the general practitioner training system, and improving service capacity to build public trust. On the policy side, reforms should encourage digitalization, enhance personalized health management, expand prescription circulation and telemedicine, and strengthen preventive and rehabilitation services through family doctor contracts and community-based care.

#### 4.2.4 Building a multi-stakeholder collaborative health governance pattern

With China's social and economic development, health improvement should no longer be limited to medical institutions. Families, communities, schools, enterprises, and social organizations should all play active roles. The key to better health lies not only in financial investment or infrastructure, but in disease prevention. The government should guide these entities to take responsibility for public health by promoting health education and preventive knowledge in daily life. Through multi-sectoral collaboration, promote the strategic transformation from “treatment-centered” to a “health-centered” approach, improve national health literacy, and advance the goal of universal health.

### 4.3 Limitations

This study used self-rated health as a measure for health status. While self-rated health is a widely used indicator, it is inherently subjective and may be influenced by individual psychological states, cultural backgrounds, and other factors, potentially introducing measurement errors. Although the PSM and IV methods were used to mitigate the endogeneity concerns, the selected IV “job type” might not fully satisfy the exogeneity assumption. Specifically, job type could directly influence self-rated health through pathways such as income level and occupational risk, in addition to its indirect effect via health insurance. Furthermore, the logit model assumes a linear relationship between variables, whereas the association between self-rated health and health insurance may exhibit non-linear or interactive effects. For instance, different income groups may respond differently to health insurance benefits, a nuance that the current model does not fully capture. Additionally, the dataset spans only two time periods (2018 and 2020), which limits the analysis of long-term effects of health insurance, such as cumulative effects of prolonged enrollment on health outcomes, or the impact of dynamic policy adjustments over time.

Although social health insurance is a national initiative aimed at address health issues through institutional arrangements, this study highlights that the effect of URRBMI on improving self-rated health among the insured has fallen short of expectations. Beyond enhancing healthcare utilization rates, it is crucial to actively promote positive health behaviors, including active health management, preventive measures, and healthy lifestyle choices. Future research should aim to uncover the complex mechanisms underlying the health insurance system's impact on health by employing additional comprehensive health indicators, multidimensional data, mixed-methods approaches, and detailed policy comparisons.

## Data Availability

The data analyzed in this study is subject to the following licenses/restrictions: The data that support the findings of this study are available from Peking University, but restrictions apply to the availability of these data, which were used under license for the current study and are not publicly available. However, the data are available from the authors upon reasonable request and with the permission of Peking University. Requests to access these datasets should be directed to https://opendata.pku.edu.cn/dataverse/CFPS.
